# Novel Spectrophotometric Method for the Assay of Captopril in Dosage Forms using 2,6-Dichloroquinone-4-Chlorimide

**Published:** 2008-06

**Authors:** Nahed El-Enany, Fathalla Belal, Mohamed Rizk

**Affiliations:** 1 *Department of Analytical Chemistry, Faculty of Pharmacy, University of Mansoura, Mansoura, Egypt;*; 2 *Department of Analytical Chemistry, Faculty of Pharmacy, University of Helwan, Cairo, Egypt*

**Keywords:** captopril, 2,6-dichloroquinone-4-chlorimide (DCQ), spectrophotometry, dosage forms

## Abstract

A simple spectrophotometric method was developed for the determination of captopril (CPL) in pharmaceutical preparations. The method is based on coupling captopril with 2,6-dichloroquinone-4-chlorimide (DCQ) in dimethylsulphoxide. The yellow reaction product was measured at 443 nm. The absorbance–concentration plot was rectilinear over the range of 10-50 μg/mL with minimum detection limit (LOD) of 0.66 μg/mL and a quantification limit (LOQ) of 2.0 μg/mL. The different experimental parameters affecting the development and stability of the color were carefully studied and optimized. The proposed method was successfully applied to the analysis of commercial tablets and the results were in good agreement with those obtained using official and reference spectrophotometric methods. Hydrochlorothiazide which is frequently co-formulated with CPL did not interfere with the assay. A proposal of the reaction pathway was presented.

## INTRODUCTION

**Figure 1:**
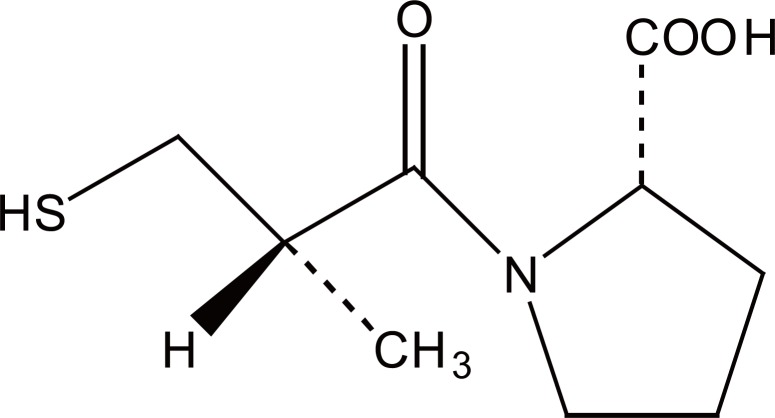
Structural formula of captopril.

Captopril (CPL) (Fig 1), 1-(3-mercapto-2-D-methyl-1-oxoproppyl)-l-proline (S,S), is used therapeutically as an antihypertensive agent. It acts as a potent and specific inhibitor of angiotensin-converting enzyme (1). It is used in the management of hypertension, in heart failure, following myocardial infraction and in diabetic nephropathy.

Several methods have been reported for the quantitative determination of captopril in formulations and biological fluids. A method based on the oxidation of CPL with excess potassium permanganate and measuring the remaining drug using different dyes was reported (2). Also, CPL was determined through complex formation with palladium (3), through reaction with carbon disulphide (4), and through reaction with dinitrobenzene (DNB) (5), nitrous acid and cresyl fast violet (6) and benzene sulphonyl chloride (BSC) (7), GC-MS (8-9), HPLC (10-19), electrochemistry (20-24), chemiluminescence (25), and capillary electrophoresis (26). The official USP method recommends titration of captopril with potassium iodate in acidic medium (27).

2,6-Dichloroquinone-4-chlorimide (DCQ) has been frequently utilized as an analytical reagent in pharmaceutical analysis. It has been used for the colorimetric determination of certain sympathomimetic drugs (28), prenalterol at 595 nm (29), thiol compounds such as penicillamine at 431 nm (30) and amines containing drugs viz sodium floxacillin (31) and leflunomide (32).

The aim of the present approach was to to develop a simple spectrophotometric method for the accurate and rapid analysis of CPL using DCQ as a coloring reagent. Moreover, no interference was encountered from the commonly co-formulated drug, hydrochlorothiazide. Another advantage of the proposed method as compared to other existing ones is that it is simple, rapid and requires no prior extraction step.

## EXPERIMENTAL

### Materials

CPL was kindly supplied by Squibb Egypt Co. Giza, Egypt. Pharmaceutical preparations including: capozide tablets containing 50 mg of captopril and 25 mg of hydrochlorothiazide each (Batch # E11477); product of Squibb Egypt Co. Giza, Egypt and capoten tablets containing 25 mg of captopril each (Batch # B10401204) product of Squibb Egypt Co. Giza, Egypt.

All pharmaceutical preparations were obtained from commercial sources in the local market.

### Reagents

All the reagents were of Analytical Reagent Grade. 2,6-dichloroquinone-4-chlorimide (DCQ) was obtained from Koch-light laboratories (England), a 0.25% w/v solution was freshly prepared in ethanol. Dimethylsulphoxide (DMSO) was obtained from Nentech Ltd (NTL) Brixworth-Northants (UK). Acetate buffer 0.2 M (pH5.0) was prepared by mixing appropriate volumes of 0.2 M acetic acid with 0.2 M sodium acetate. Borate buffers (pH7 and 9) were prepared by mixing appropriate volumes of 0.02 M boric acid with 0.2 M sodium hydroxide. Ammonia buffer (pH10) was prepared by mixing appropriate volumes of 0.2 M ammonium chloride with 0.2 M ammonium hydroxide (33).

### Apparatus

UV- VIS 1601, Shimadzu recording Spectrophotometer (P/N 206-67001). Recording range from 0 to1.0, wavelength 443 nm.

### Standard Solutions

A Stock solution was prepared by dissolving 20.0 mg of CPL in 100 mL of distilled water and was further diluted with the same solvent as appropriate. The standard solutions were stable for one week when kept in the refrigerator.

## METHOD

### Recommended procedures

**Calibration curve.** Aliquot volumes of CPL covering the working concentration range (10-50 μg/mL) were transferred into a series of 10 ml volumetric flasks. 5 mL ± 1 mL of DMSO was added, followed by addition of 0.7 ± 0.1 mL of DCQ. The flasks were completed to the mark with distilled water and allowed to cool. The absorbance of the resulting solution was measured at 443 nm against a reagent blank prepared simultaneously. The values of the absorbance were plotted against the final concentration of the drug in μg/mL. Alternatively, the corresponding regression equation was derived.

### Optimization of the reaction conditions

**Influence of pH.** Aliquot volumes of CPL 30 μg/mL was transferred into a series of 10 ml volumetric flasks. 5 ± 1 mL of DMSO was added, followed by 2 mL of acetate buffer (pH5) or borate buffer (pH7 or 9), or 0.1 M NaHCO_3_ (pH8) or 2 mL of ammonia buffer (pH10.0). 0.7 ± 0.1 mL of DCQ were then added. The flasks were completed to the mark with distilled water and allowed to cool. The absorbance of the resulting solutions was measured at specified wavelength (nm) cited in Table 1 against a reagent blank prepared simultaneously.

**Table 1:** Effect of pH on the formation, intensity and stability of the reaction product of captopril (30 μg/mL)

Buffer (pH)	λ max. (nm)	Absorbance	Stability

No buffer	443	0.355	Stable
Acetate buffer (5)	415	0.23	Stable
Borate buffer (7)	439	0.277	Stable
0.1 M NaHCO_3_ (8)	443	0.210	Stable
Borate buffer (9)	448	0.289	Not stable
Ammonium buffer (10)	440	0.301	Not stable

**Influence of different solvents.** Aliquot volumes of CPL 30 μg/mL was transferred into a series of 10 ml volumetric flasks. 5 ± 1 mL DMS or any of the studied solvent as cited in Table 2, followed by 0.7 ± 0.1 mL of DCQ. The flasks were completed to the mark with distilled water and allowed to cool. The absorbance of the resulting solutions was measured at specified wavelength (nm) cited in Table 2 against a reagent blank prepared simultaneously.

**Table 2:** Effect of different solvents on the color formation and intensity of the reaction product of captopril (30 μg/mL)

Solvent	Color development	λ max (nm)	Absorbance

Acetonitrile	Very slow	435	0.056
Acetone	Slow	437	0.195
Methanol	Slow	434	0.182
Ethanol	Slow	437	0.222
Water	Immediate	411	0.168
Dimethylsulphoxide	Immediate	443	0.355

**Influence of surfactants and sensitizers.** Aliquot volumes of CPL 30 μg/mL were transferred into a series of 10 ml volumetric flasks. 5 ± 1 mL of DMSO was added, followed by 0.7 ± 0.1 mL of DCQ. Different surfactants (cetrimide, gelatin and sodium lauryl sulfate) at three different concentrations (2.5, 7.5 and 15 μg/mL) were added to the reaction mixture before measuring the absorbance values of the reaction product. Similarly, different sensitizers such as quinine, fluorescein and rhodamine-B, at concentrations of 5 μg/mL were added. The flasks were completed to the mark with distilled water and allowed to cool. The absorbance of the resulting solutions were measured at 443 nm against a reagent blank prepared simultaneously (Table 3).

**Table 3:** Effect of surfactants and sensitizers on the absorbance of the reaction product of captopril (30 μg/mL) with DCQ

Substance	Concentration, μg/mL	Absorbance

No surfactant	–	0.355
Cetrimide	2.5	0.302
Sodium lauryl sulfate	2.5	0.351
Gelatin	2.5	0.347
Cetrimide	7.5	0.307
Sodium lauryl sulfate	7.5	0.416
Gelatin	7.5	0.327
Cetrimide	15	0.325
Sodium lauryl sulfate	15	0.442
Gelatin	15	0.337
No sensitizer	–	0.355
Quinine	5	0.348
Fluorescein	5	0.385
Rhodamine-B	5	0.404

### Applications

**Procedure for tablets.** Twenty tablets were weighed and pulverized. A weighed quantity of the powder equivalent to 20.0 mg of CPL was transferred into a small conical flask, extracted with 3 × 30 mL of distilled water. The extract was filtered into 100 mL volumetric flask. The conical flask was washed with water, passed into the same volumetric flask and completed to the mark with the same solvent. Aliquot volumes covering the working concentration range (cited in Table 4) was transferred into 10 mL volumetric flasks. The recommended procedures under calibration curve were then performed. The nominal content of the tablets were determined either from the calibration curve or using the corresponding regression equation.

**Table 4:** Performance data of the proposed method

Parameter	Proposed method

Concentration range, μg/mL	10-50
Limit of detection (LOD) (μg/mL)	0.66
Limit of Quantification (LOQ) (μg/mL)	2.0
Correlation coefficient (r).	0.9997
Slope	0.012
Intercept	1.8 × 10^-3^
S _y/x_	5.45 × 10^-3^
S_a_	2.33 × 10^-3^
S_b_	1.72 × 10^-4^
%Error	0.52
Applications	Tablets

S_y/x_, standard deviation of the residuals; S_a_, standard deviation of the intercept of regression line; S_b_, standard deviation of the slope of regression line; % Error, RSD% / √ n.

## RESULTS AND DISCUSSION

**Figure 2:**
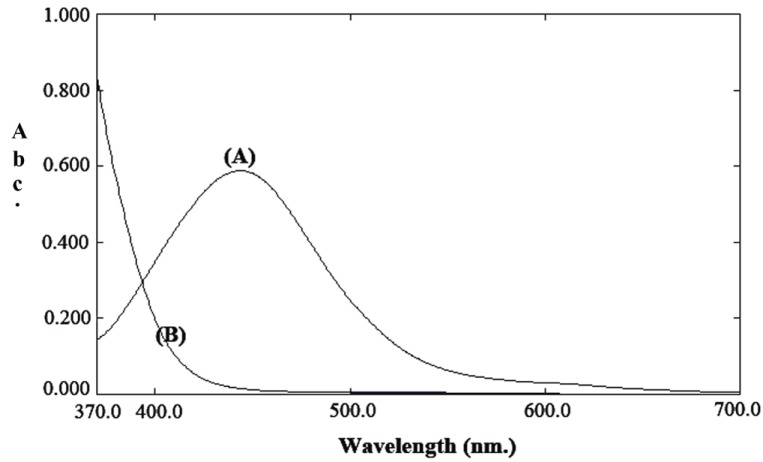
Absorption spectra of: **A,** the reaction product of captopril (50 μg/mL) and DCQ; **B,** reagent blank.

Captopril contains a thiol group which is susceptible for the reaction with DCQ and produces a yellow color that peaked at 443 nm (Fig. 2). The spectrophotometric properties of the colored product and the different experimental parameters affecting the color development and its stability were carefully studied and optimized. Such factors were changed individually while others were kept constant. The factors include pH, type of buffer, effect of different solvent, concentration of the reagent, effect of sensitizers and surfactants. All the studies were carried out at room temperature (25°C).

### Optimization of Reaction condition

**Effect of pH.** The influence of pH on the absorbance value of the reaction product was evaluated. Reactions were carried out at different pH values ranging from pH5.0 to pH9.0. Maximum color intensity was obtained using either borate buffer (pH9.0) or ammonia buffer at pH10. However the developed color in these media was unstable. The color developed in other buffers such as acetate (pH5.0), borate (pH7.0) and sodium bicarbonate (pH8.0) was stable but had low absorbance values (Table 1). Additionally, it was found that changing the pH values resulted in a subsequent change in the maximum wavelength of the reaction product. This result may be attributed to the release of proton.

**Effect of different solvents.** The effect of different solvents, i.e. acetonitrile, acetone, methanol, ethanol, water and DMSO, on the color development was studied. Immediate and stable color was developed using DMSO as compared with the other solvents. The results are abridged in Table 2. The observed heat released upon addition of DMSO to the aqueous solution of the drug (exothermic reaction) and the change in the viscosity of the solution gave optimal conditions for the reaction of the drug with the reagent. This is confirmed by finding that the sequence of addition of the reagent was essential to obtain maximum color intensity. Solutions were therefore allowed to cool before measuring absorbance values of the reaction products. The lowest color intensity was observed using acetonitrile.

**Figure 3:**
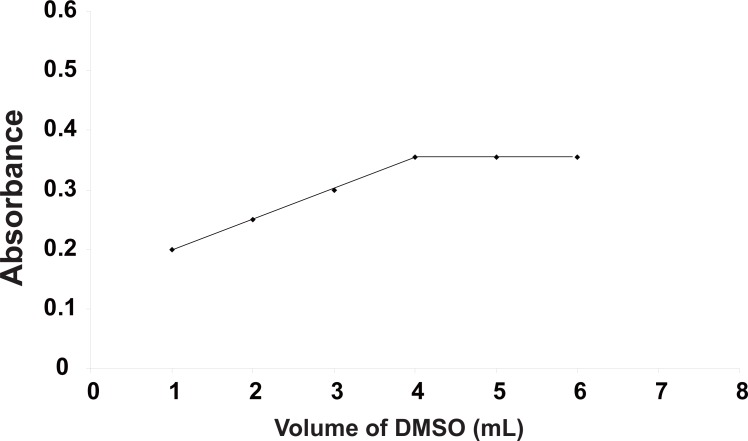
Effect of volume of DMSO (mL) on the absorbance value of the reaction product (captopril 30 μg/mL) at 443 nm.

**Effect of volume of DMSO.** The effect of volume of DMSO on the color intensity was studied. Increasing the volume of DMSO resulted in a subsequent increase in the absorbance intensity of the reaction product up to 4 mL and remained constant until 6 mL. Thus, 5 ± 1 mL was chosen as the optimum volume of DMSO (Fig. 3).

**Figure 4:**
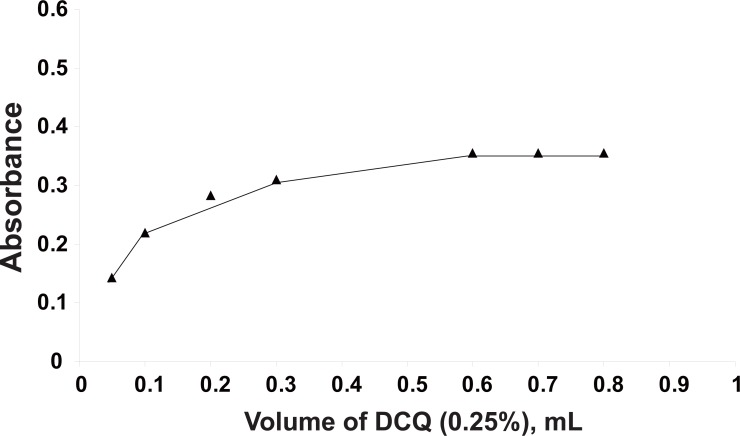
Effect of volume of DCQ (0. 25%) (mL) on the absorbance value of the reaction product (captopril 30 μg/mL) at 443 nm.

**Effect of volume of DCQ.** The optimum volume and concentration for the DCQ reagent required was found to be 0.7 ± 0.1 mL of 0.25% w/v (Fig. 4).

**Effect of surfactants and sensitizers.** The effect of surfactants and sensitizers on the color development was also studied. Different surfactants (cetrimide, gelatin and sodium lauryl sulfate) at three different concentrations (2.5, 7.5 and 15 μg/mL) were added to the reaction mixture before measuring the absorbance value of the reaction product. The results are shown in Table 3. It is clear that cetrimide and gelatin resulted in a slight decrease in the absorbance value of the reaction product. On the other hand, sodium lauryl sulfate promoted a slight increase the absorbance value of the reaction product. Additionally, different sensitizers including quinine, fluorescein and rhodamine-B, at concentrations of 5 μg/mL were tested by adding to the reaction mixture before measuring the absorbance of the product. Only among them, fluorescein and rhodamine-B produced a slight increase in the absorbance value (Table 3).

### Analytical performance

**Validation of the proposed methods.** The validity of the method was tested regarding linearity, specificity, accuracy, repeatability and precision according to ICH Q2B recommendations (34).

#### a) Linearity

The absorbance-concentration plot was rectilinear over the range of 10-50 μg/mL with minimum detection limit of 0.66 μg/mL. Linear regression analysis of the data gave the following equation:

$A=1.8\times 10^{-3}+0.012C\;\left(r=0.9997\right)$

Where A is the absorbance in 1 cm cell and C is the concentration of the drug in μg/mL and r is correlation coefficient.

The limit of quantification (LOQ) was determined by establishing the lowest concentration that can be measured according to ICH Q2B (34). The results are shown in Table 4. The limits of detection (LOD) were determined by establishing the minimum level at which the analyte can be reliably detected, and the results are also abridged in Table 4.

LOQ and LOD were calculated according to the following equation (34):

LOQ= 10 σ /S

LOD= 3.3 σ /S

where σ: the standard deviation of the intercept of regression line. S: Slope of the calibration curve.

The proposed methods were evaluated for the accuracy as percent relative error (% Er) and the precision as percent relative standard deviation (% RSD) (Table 4).

**Accuracy and precision.** The reproducibility or precision of the method was evaluated by statistical analysis of the regression data regarding standard deviation of the residuals (S_y/x_), the intercept (S_a_) and the slope (S_b_). The small values of the figures point out to the low scattering of the calibration graph and high precision.

Statistical analysis (35) of the results, obtained by the official (27) and the proposed method using Student’s t-test and Variance ratio F-test, show no significant difference between the performance of the two methods regarding the accuracy and precision, respectively (Table 5).

**Table 5:** Application of the proposed method and official methods for the determination of captopril in pure form

Parameters	Proposed method	Official method (27)

No. of experiments	5	3
Mean found, % ± SD	100.15 ± 1.17	99.89 ± 0.78
RSD, %	1.17	0.78
Variance	1.37	0.61
Student’s t-value	0.34 (2.45)	-
Variance ratio F-test	2.25 (6.94)	-

Values in parentheses are the tabulated values of t and F respectively at p = 0.05 (35).

#### b) Accuracy

To test the accuracy of the proposed method an amount of authentic CPL (30 μg/mL) was added to capozide tablets (20 μg/mL) and subsequently assayed by the proposed method. The mean percentage recovery of the added quantity was found to be 100.40 ± 0.50. This indicates that the proposed method gives accurate results.

#### c) Precision

**Repeatability.** To test the reproducibility of the proposed method, six replicate analyses were carried out for a concentration of 50 μg/mL of CPL. The mean percentage recovery was found to be 100.55 ± 0.54.

**Specificity.** The specificity of the method was investigated by observing any interference encountered from the excipients of tablets. Hydrochlorothiazide which is frequently co formulated with captopril did not interfere with the proposed method (Table 6).

**Table 6:** Application of the proposed method to the determination of captopril in dosage forms

Preparation	% Recovery Proposed method	% Recovery Reference method (6)

1-Capoten tablets^a^ (CPL, 25 mg/ tablet)	98.73	99.64
	100.83	100.54
	99.88	101.30
	98.40	-
	99.15	-
X^-^ ± SD.	99.40 ± 0.97	100.49 ± 0.83
t-value.	1.61(2.45)	-
F-value	1.36 (6.94)	-
2-Capozide tablets^b^ (CPL, 50 mg and 25 mg of hydrochlorothiazide/ tablet)	100.32	99.50
	100.41	101.05
	100.28	100.88
	98.84	-
	99.83	-
X^-^ ± SD.	99.94 ± 0.65	100.48 ± 0.85
t-value.	1.03 (2.45)	-
F-value.	1.71 (6.94)	-

a product of Squibb Egypt Co. Giza, Egypt (Batch # B10401204);

b product of Squibb Egypt Co. Giza, Egypt (Batch # E11477).

Values in parenthesis are the tabulated; t and F values respectively at p = 0.05 (35).

#### d) Robustness of the method

The robustness of the method adopted is demonstrated by the constancy of the absorbance with the deliberated minor changes in the experimental parameters such as volume of DMS, 5 ± 1 mL, and change in the volume of DCQ (0.25%), 0.7 ± 0.1 mL. These minor changes that may take place during the experimental operation did not affect the absorbance of the reaction product.

### Pharmaceutical applications

The proposed method was applied to the determination of CPL in tablets. Common tablet excipients such as talc, lactose, starch, avisil, gelatin and magnesium stearate did not interfere with the assay. Statistical analysis (35) of the results, obtained by the proposed and the reference (6) methods using Student’s t-test and Variance ratio F-test, show no significant difference between the performance of the two methods regarding the accuracy and precision, respectively (Table 6).

Upon degradation of CPL with 0.05 M iodine solution (26), the main degradation product, which is reported to be captopril disulphide, did not interfere with the assay as revealed by no absorption peak upon addition of DCQ.

### Mechanism of the reaction

**Figure 5:**
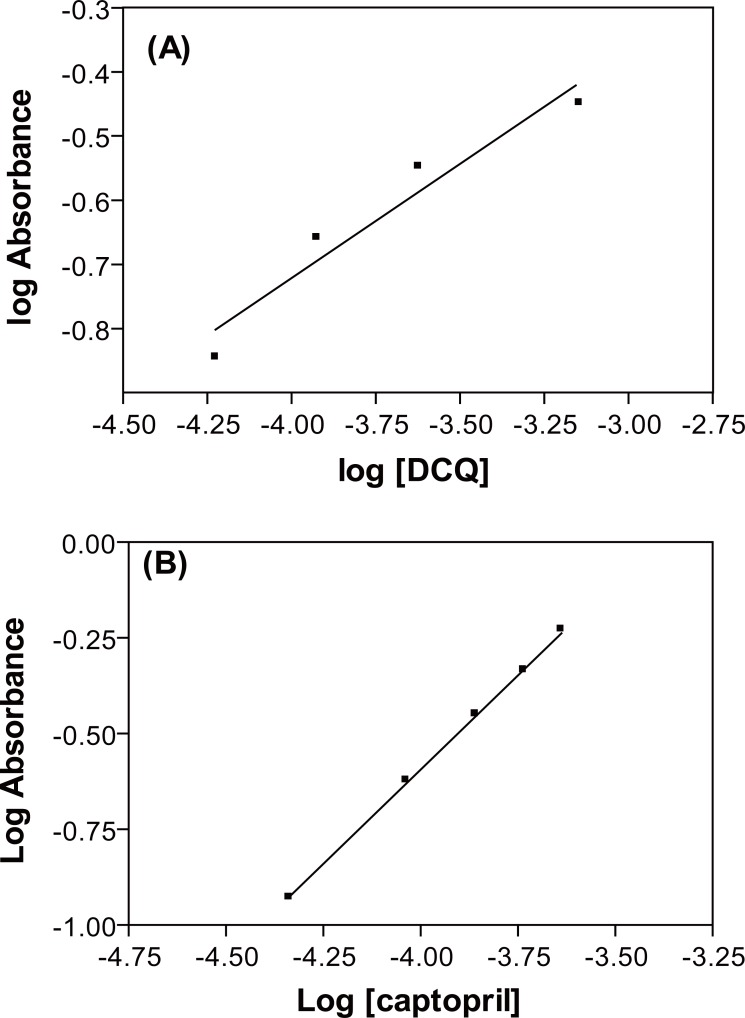
Limiting logarithmic plots for the molar ratio. (**A**) Log A *vs.* Log (DQ); (**B**) Log A *vs.* log (captopril).

**Figure 6:**
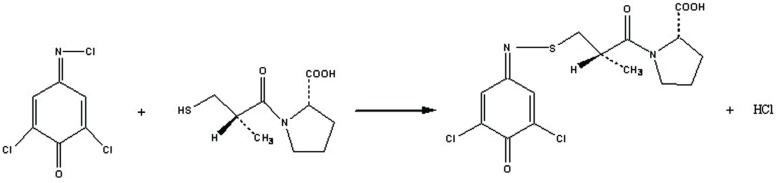
Proposal of the reaction pathway between DCQ and captopril.

The stoichiometry of the reaction was studied adopting the limiting logarithmic method (36). The absorbance of the reaction product was alternatively measured in the presence of excess of both DCQ and CPL. A plot of log absorbance versus log (DCQ) and log (CPL) gave straight lines; the values of the slopes were 0.64 and 0.99, respectively. Hence it is concluded that the molar reactivity of the reaction is 0.64/0.99, i.e., the reaction proceeds in the ratio of 1:1. A schematic proposal of the reaction pathway is shown in Figure 5 and Figure 6.

## CONCLUSION

The proposed method is accurate, precise and simple. 2,6-dichloroquinone-4-chlorimide (DCQ) proved to be a suitable reagent for the determination of CPL in pure form and its dosage forms. The proposed method is simple, time saving (the analysis of one sample takes less than 5 min.) and reproducible. The suggested method can be used for the determination of CPL in quality control and industry.
